# Prevalence of antidepressant use in Brazil: a systematic review with meta-analysis

**DOI:** 10.47626/1516-4446-2023-3095

**Published:** 2024-03-25

**Authors:** Gustavo Magno Baldin Tiguman, Rogério Hoefler, Marcus Tolentino Silva, Vanessa Gomes Lima, Inês Ribeiro-Vaz, Taís Freire Galvão

**Affiliations:** 1 Faculdade de Ciências Farmacêuticas, Universidade Estadual de Campinas, Campinas, SP, Brazil; 2 Departamento de Medicina da Comunidade, Informação e Decisão em Saúde, Faculdade de Medicina, Universidade do Porto, Porto, Portugal; 3 Departamento de Saúde Coletiva, Faculdade de Ciências da Saúde, Universidade de Brasília, Brasília, DF, Brazil; 4 Unidade de Farmacovigilância do Porto, Centro de Investigação em Tecnologias e Serviços de Saúde, Faculdade de Medicina, Universidade do Porto, Porto, Portugal

**Keywords:** Antidepressive agents, drug utilization, mental health, prevalence, systematic review

## Abstract

**Objectives:**

To estimate the prevalence of antidepressant use in Brazil.

**Methods:**

We conducted a systematic review with searches in MEDLINE, Embase, Scopus, LILACS, and SciELO up to May 2023. Two researchers independently selected studies, extracted data, and assessed the methodological quality. We pooled the prevalence of antidepressant use using meta-analyses of proportions (Freeman-Tukey transformation) and estimated heterogeneity by the *I* ^2^ statistic. OR meta-analyses of antidepressant use by sex were calculated (men as reference) and between-study variation was explored by meta-regressions.

**Results:**

Out of 3,299 records retrieved, 23 studies published in 28 reports were included, with a total of 75,061 participants. The overall prevalence of antidepressant use was 4.0% (95%CI 2.7-5.6%; *I*^2^ = 98.5%). Use of antidepressants in the previous 3 days was higher in women (12.0%; 95%CI 9.5-15.1%; *I*^2^ = 0%) than men (4.6%; 95%CI 3.1-6.8%; *I*^2^ = 0%) (p < 0.001; OR = 2.82; 95%CI 1.72-4.62). Gender differences were particularly higher for antidepressant use in the previous year (women: 2.3%; 95%CI 1.6-3.1; *I*^2^ = 37.6% vs. men: 0.5%; 95%CI 0.2-1.0%; *I*^2^ = 0%, p < 0.001; OR = 4.18; 95%CI 2.10-8.30). Between-study variation in the overall prevalence of antidepressant use significantly increased with mean participant age (p = 0.035; residual *I*^2^ = 0%; regression coefficient = 0.003).

**Conclusion:**

Four out of every 100 Brazilians used antidepressants in this 3-decade assessment. Use increased with age and was more prevalent in women compared to men.

**Registration number:**

PROSPERO CRD42022345332.

## Introduction

The burden of mental disorders is increasing worldwide, with no evidence of any reduction since 1990.^1^ Poverty, unemployment, social disparities, and cultural factors may contribute to this high burden in low- and middle-income countries (LMIC), where investment in mental health services and access to appropriate pharmacological treatments are limited.^2^,^3^ Specifically in Brazil, profound changes in urbanization which increased the proportion of the population living in peripheral and underserved areas, successive economic crises, deregulation of working conditions and erosion of labor laws have been pointed out as factors associated with the high rates of mental illness in the country, in addition to individual socioeconomic, behavioral, and health factors.^4^,^5^

Antidepressants are medicines commonly used to treat such conditions. Sales of antidepressant drugs in Brazil have increased over time, mainly driven by high prescription of newer therapeutic classes.^6^,^7^ Although data related to trends in drug sales are available, recent estimates on the proportion of the Brazilian population under antidepressant treatment are lacking. Investigation of the prevalence of antidepressant use in the community setting may serve as an important indicator of access to mental health care, especially in vulnerable settings.^3^ Previous population-based surveys on the prevalence of antidepressant use have been conducted in different regions of Brazil, but no summary of these results considering only studies with representative samples is available. Thus, the aim of this systematic review was to estimate the prevalence of antidepressant use in the Brazilian population.

## Methods

The protocol of this systematic review, describing the methods applied in detail, was registered in the International Prospective Register of Systematic Reviews (CRD42022345332) and is available at: www.crd.york.ac.uk/prospero/display_record.php?ID=CRD42022345332.

### Eligibility criteria

Population-based studies with representative samples that have investigated the prevalence of antidepressant use among adults (≥ 18 years old) in Brazil were eligible for inclusion. The question framework was as follows: Population – Brazilian adults; Outcome – Prevalence of antidepressant use; Study type – Population-based, representative studies. We considered as “population-based” those studies that were conducted in the community, with the general population (studies held in health care settings, for instance, were not eligible). Samples were considered representative if studies included participants selected by a probabilistic sampling process. Antidepressant drugs were selected as reported in the primary studies, with definitions derived from international drug coding systems or national medicine formularies. No restrictions on language or publication dates were applied.

### Information sources and search strategy

Searches were performed in May 2022 and fully updated in May 2023 in the following databases: PubMed, Embase, Scopus, LILACS, and SciELO.

The full search strategies for all databases are described in Table S1, available as online-only supplementary material. The Peer Review of Electronic Search Strategies guidance^8^ was followed to review the pilot strategy developed for PubMed, which was then adapted for the other databases. Search results for all databases were imported into the Covidence platform (www.covidence.org) to remove duplicates and further perform study selection, data extraction, and methodological quality appraisal.

The references of relevant publications were also screened for evaluation of potentially eligible studies.

### Selection process

Two researchers (GMBT and RH) independently selected studies by screening titles and abstracts. Calibration of the selection was performed with 100 studies. Based on discrepancies in this pilot phase, consensus meetings were held for refinement of the selection process and clarification of questions related to the eligibility criteria. The full text of studies that potentially met the eligibility criteria was then assessed using the Covidence platform. Any disagreements were resolved by consensus with a third reviewer (TFG).

### Data collection process

Two independent authors (GMBT and RH) initially participated in a pilot extraction of two studies. Disagreements were again resolved in a consensus meeting to calibrate the data extraction process and adjust the data collection form. Data extraction was performed by two independent reviewers (GMBT and RH) and confirmed by a third one (TFG) using a standardized spreadsheet uploaded into the Covidence platform. Disagreements were resolved by consensus among the three reviewers. If additional data or any clarifications from the selected studies were required, we attempted to contact the study authors to request information.

### Data items

The following data were collected: study data (author, publication date, data collection date, location, study design, and sampling method), sample characteristics (eligibility criteria, sample size, and age), number of participants on antidepressants out of the total adult population stratified by sex (men, women) and age group (adults [≥ 18 years old, including older adults], older adults [≥ 60 years old]), and total number of participants assessed. Antidepressant data included the prevalence of antidepressant use, recall period of medicine use (timeframe specified during the interviews for prior medicine use), if confirmation of medical prescriptions or drug packages was performed during the survey (yes, no), and coding system used for the classification of medicines (e.g., World Health Organization Anatomical Therapeutic Chemical [ATC] classification system).

### Study quality assessment

Two independent researchers (GMBT and RH) assessed the methodological quality of the selected studies using the Joanna Briggs Institute checklist for prevalence studies.^9^ A third reviewer (TFG) independently confirmed the ratings. Disagreements were resolved by consensus. We assessed the quality using the nine items of the instrument: 1) sample frame; 2) recruitment of participants; 3) adequate sample size; 4) adequate description of participants; 5) appropriateness of data coverage; 6) valid methods for outcome measurement; 7) standardization of outcome measurement for all participants; 8) statistical analysis properly performed and reported; 9) response rate. Table S2 (available online-only) details the criteria adopted to judge each item. We assessed each domain as either “yes” (1), if the criterion was fulfilled, or “no” (0), if it did not or only partially satisfied the item. Therefore, the maximum score was 9 points per study.

### Effect measures

The primary outcome was the prevalence of antidepressant use in Brazil with 95%CI.

### Synthesis methods

Meta-analyses of proportions were calculated by the Freeman-Tukey double arcsine transformation^10^ in Stata 14.2 (*metaprop* command, *ftt* option). Subgroup analyses were performed for recall period, sex, and age, while differences in prevalence were assessed by the Cochran’s Q test and associated p-values. Meta-analysis of the odds ratio (OR) of antidepressant use by sex (odds of antidepressant use in women divided by the odds in men) was calculated using the DerSimonian & Laird method (*metan* command). Random effects were considered in all meta-analyses and heterogeneity was assessed by the inconsistency between studies (*I*^2^).

Meta-regressions were calculated by the modified Knapp-Hartung method^11^ to assess the effect of participants’ mean age, the start and end years of the survey, and the recall period on the variability of antidepressant use prevalence between studies.

Subgroup analyses for study region and dates were initially planned in the protocol, but were not conducted after data collection, since only two studies were conducted outside the South and Southeast regions and meta-regressions showed study dates had no influence on prevalence variability.

### Reporting bias assessment

Reporting bias was assessed by visual inspection of the funnel plot asymmetry and Egger’s test^12^ (p < 0.05 deemed significant).

## Results

### Study selection and characteristics

**Figure 1:**
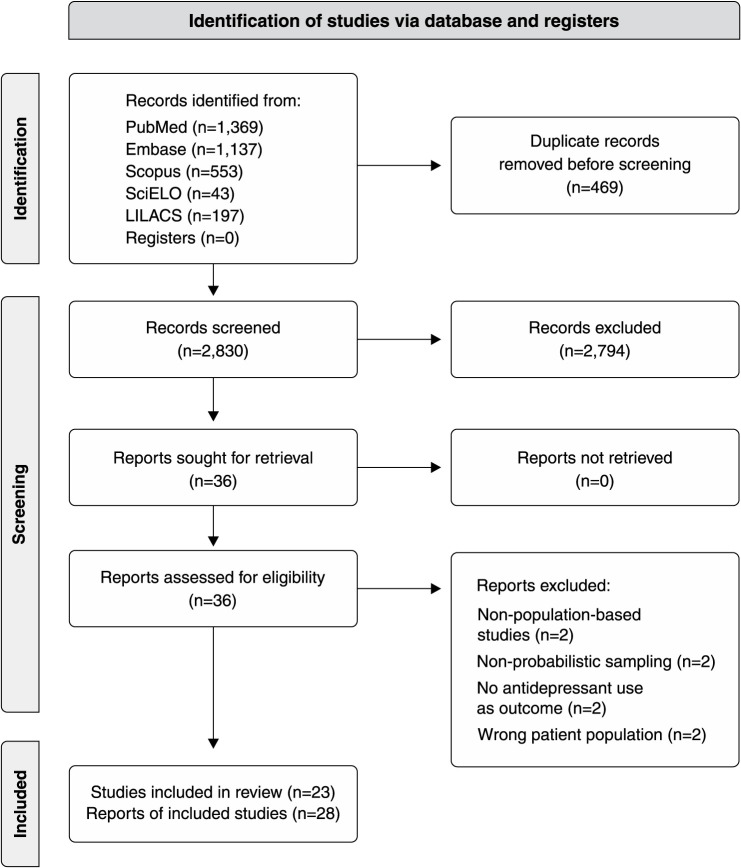
Flow diagram of study selection and inclusion.

Out of the 3,299 publications retrieved from the search, 23 studies published in 28 reports were included^13^-^35^ (Figure 1). In total, 75,061 individuals aged ≥ 18 years were assessed in surveys conducted from 1990 to 2021. Fifteen studies were conducted in the Southeast region of Brazil,^13^,^15^-^17^,^20^-^23^,^27^-^31^,^35^,^36^ five in the South,^14^,^18^,^19^,^24^,^32^ two in the North,^26^,^34^ and one had nationwide coverage.^33^ Three were cohort studies^4^,^21^,^35^ and the remainder had cross-sectional designs. The recall period to measure the use of antidepressants ranged from the day of the interview (0 days) to 1 year prior. Age extremes were included in six studies; one survey considered participants aged ≥ 14 years, and five included older adults (age ≥ 60 years) (Table 1).

### Methodological quality of studies

Adequate sample sources (22/23) and sampling processes (23/23) were the highest-rated items on methodological quality assessment, while confirmation of outcomes by checking medical prescriptions or drug packages and/or classification of medicines using a coding system (12/23) and appropriate statistical analysis with presentation of numerators and denominators and/or measures of dispersion (12/23) were the lowest-scoring items among the included studies (Table S3, available as online-only supplementary material).

### Results of syntheses

#### Prevalence of antidepressant use

**Figure 2:**
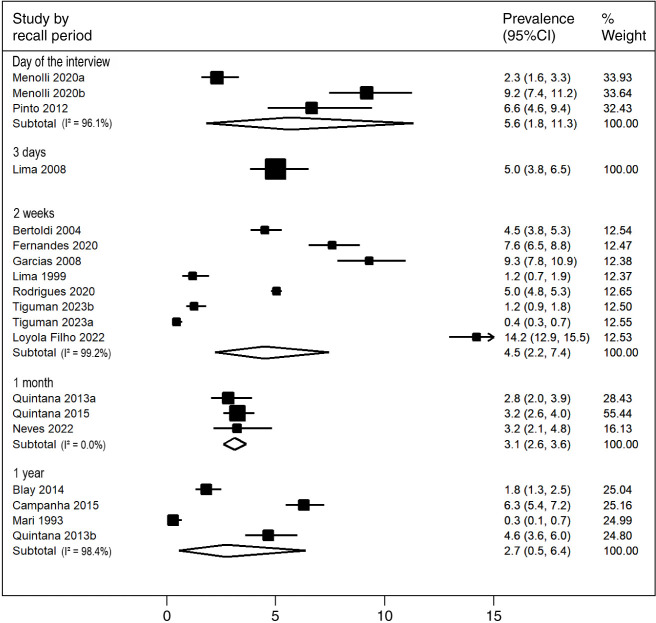
Prevalence of antidepressant use (%) and 95%CI according to the recall period adopted.

The overall prevalence of antidepressant use was 4.0% (95%CI 2.7-5.6%; *I*^2^ = 98.5%). Antidepressant use was assessed for the previous 15 days in eight studies, with prevalence estimates of 4.5% (95%CI 2.2-7.4%; *I*^2^ = 99.2%) in adults. The highest prevalence was 5.6% (95%CI 1.8-11.3%; *I*^2^ = 96.1%), observed in the population that reported using antidepressants on the day of the interview, followed by a prevalence of 5.0% (95%CI 3.8-6.5%; *I*^2^ = 0%) in the previous 3 days. Lower prevalence of antidepressant use was found for the past 30 days (3.1%; 95%CI 2.6-3.6%; *I*^2^ = 0%) and for the previous year (2.7%; 95%CI 0.5-6.4%; *I*^2^ = 98.4%) (Figure 2).

#### Prevalence of antidepressant use according to sex and age groups

Antidepressant use in the previous 3 days was higher in women (12.0%; 95%CI 9.5-15.1%; *I*^2^ = 0%) compared to men (4.6%; 95%CI 3.1-6.8%; *I*^2^ = 0%), p < 0.001; OR = 2.82 (95%CI 1.72-4.62). The same results were observed for all the remaining recall periods: 15 days (women: 4.6%; 95%CI 0.0-19.5%; *I*^2^ = 0% vs. men: 2.1%; 95%CI 0.0-7.4%; *I*^2^ = 0%; p = 0.638; OR = 2.22; 95%CI 1.32-3.73), 30 days (women: 4.3%; 95%CI 3.5-5.2%; *I*^2^ = 0% vs. men: 1.1%; 95%CI 0.6-1.7%; *I*^2^ = 0%; p < 0.001; OR = 4.02;95%CI 2.42-6.70), 90 days (women: 11.4%; 95%CI 9.5-13.6%; *I*^2^ = 0% vs. men: 3.9%; 95%CI 2.7-5.7; *I*^2^ = 0%; p < 0.001; OR = 3.17; 95%CI 2.03-4.95), and 360 days (women: 2.3%; 95%CI 1.6-3.1; *I*^2^ = 37.6% vs. men: 0.5%; 95%CI 0.2-1.0%; *I*^2^ = 0%; p < 0.001; OR = 4.18; 95%CI 2.10-8.30) (Table 2 and Figure S1, available as online-only supplementary material).

Point prevalence of antidepressant use among older adults was higher than among adults on the day of the interview (older adults: 12.2%; 95%CI 4.3-23.4%; *I*^2^ = 97.3% vs. adults: 5.6%; 95%CI 1.8-11.3%; *I*^2^ = 96.1%; p = 0.225) and in the previous 15 days (older adults: 4.6%; 95%CI 1.8-8.6%; *I*^2^ = 97.5% vs. adults: 3.5%; 95%CI 1.7-5.9; *I*^2^ = 98.9%; p = 0.590) (Table 2).

**Figure 3:**
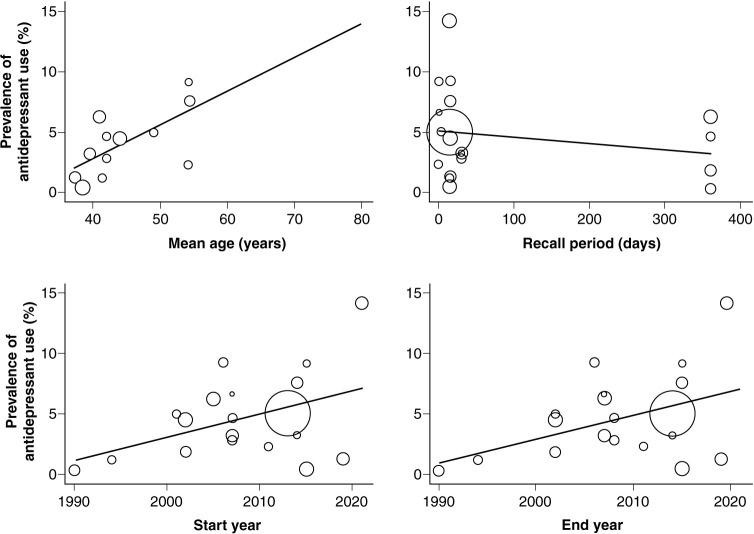
Variability of the overall prevalence of antidepressant use by mean age, recall period, and start and end years of studies.

The variability in the overall prevalence of antidepressant use was significantly affected by the participants’ mean age (p = 0.035; residual *I*^2^ = 0%; regression coefficient = 0.003), but not by the start year (p = 0.083; residual *I*^2^ = 59.6%; regression coefficient = 0.002), end year (p = 0.074; residual *I*^2^ = 59.2%; regression coefficient = 0.002), or recall period (p = 0.424; residual *I*^2^ = 65%; regression coefficient < -0.001) of the studies (Figure 3).

### Reporting biases

Visual inspection of symmetry on the funnel plot (Figure S2, available as online-only supplementary material) and Egger’s test (p = 0.001) indicated evidence of reporting biases (small-studies effect) on the prevalence of antidepressant use.

## Discussion

Nearly four out of 100 Brazilians used antidepressants in the 3-decade period covered by this systematic review. Overall, the prevalence of antidepressant use was higher in women than in men, and in older adults compared to the general adult population. Use of antidepressants increased with age, which partially explained the high variability across the studies.

Heterogeneity was an important limitation of our study, which is common in meta-analyses of prevalence.^37^ For this reason, the vast majority of meta-analyses of prevalence use random-effects modeling to obtain estimates,^38^ as we did in our study. As the primary studies were conducted in different time periods and regions, significant differences in prevalence estimates were expected. To minimize the effects of heterogeneity, we only included studies with representative samples, stratified the estimates by recall period, assessed the methodological quality of the primary studies, and conducted subgroup analyses and meta-regressions. The prevalence of antidepressant use was higher when shorter recall periods were applied when compared to longer timeframes (e.g., previous year). This may be due to memory bias, as the use of medicines was mostly assessed by self-report in the primary studies. Evidence of reporting bias was observed, as well as high heterogeneity across studies. Although these were not objectives of our systematic review, 16 out of the 23 included studies investigated diagnoses of psychiatric conditions, either by self-report or using validated tools. Data on therapeutic subgroups of antidepressants were also not collected in our review, but were reported in 12 studies, mostly as absolute numbers instead of prevalence. These data could potentially be assessed in future evidence syntheses of antidepressant use.

### Prevalence of antidepressant use

The prevalence of antidepressant use found in our study is lower than that reported by the United States National Health and Nutrition Examination Survey (NHANES) in 2018, which estimated that 13% of American adults used antidepressant drugs in the past 30 days.^39^ A similar prevalence was found in a population-based study using administrative data from Israel in 2014, which reported that 11.8% of the urban and 8.1% of the rural populations used antidepressants.^40^ A previous study conducted with 49,919 respondents from the World Health Organization World Mental Health Surveys found that the prevalence of antidepressant use was 2-4 times higher in high-income economies compared to LMIC.^41^ Lower relative personal income, higher health-related out-of-pocket costs, higher rural and urban differences, discrepancies in prescription practices, and lower availability and access to mental health services may explain these discrepancies.^3^,^41^

### Prevalence of antidepressant use according to sex and age groups

We found that antidepressant use was more prevalent among women than among men. This is consistent with the results of a population-based cross-sectional study from Sweden that included 7,725 participants in 2013, which observed that, overall, men used antidepressants to a lesser extent than women.^42^ In China, a time-trend analysis from 2013 to 2018 indicated there were nearly 1.6 times more antidepressant prescriptions for women than for men.^43^ Women tend to seek mental care and report mild-moderate depression more often than men, which can be explained by biological, behavioral, and symptomatic mechanisms.^44^,^45^

The use of antidepressants was also more prevalent in older adults than in adults in general, although this difference was not statistically significant – potentially due to the lack of statistical power of these subgroups, which is a common limitation of subgroup meta-analyses.^46^ Meta-regressions showed that the prevalence of antidepressant use increased with age. An Australian nationwide analysis of dispensing claims from 2015 to 2021 found a consistent increase in antidepressant use for women and men across most age groups, notably in individuals aged ≥ 85 years, with a 5-year change of 13.1% in women and 10.1% in men for this time period.^47^ The NHANES 2018 also found that antidepressant use increased with age, from 7.9% among individuals aged 18-39 to 14.4% for people aged 40-59 to 19% for those aged ≥ 60 years.^39^ In two English population-based cohort studies that included 7,635 people aged ≥ 65 years between 1990 and 1993 and 7,762 between 2008 and 2011, a substantial increase in the proportion of the population taking antidepressants was observed across these two decades.^48^ One plausible explanation for these findings is that a large proportion of antidepressant patients are chronic users, as observed in an Italian cohort study from 2013 that found antidepressants were mainly dispensed for long-term and chronic treatment.^49^ The use of antidepressants by older adults requires special attention, as this population may be particularly sensitive to potentially inappropriate prescriptions, especially due to polypharmacy and comorbidities.^50^,^51^

In our study, the survey year did not influence the variability in the prevalence of antidepressant use. A population-based analysis from five European settings (Sweden, Norway, Denmark, Catalonia, and Veneto) found an increasing trend in antidepressant use from 2007 to 2011.^52^ Another study conducted with a cohort of 4,030 university employees in Rio de Janeiro, Brazil, suggested that antidepressant use increased significantly over time, rising from 1.4% (1999) to 2% (2001) to 3.9% (2006-7) to 5.4% (2012).^53^

In conclusion, approximately four in every 100 Brazilians used antidepressants in this 3-decade period, a prevalence that was higher in women compared to men. The prevalence of antidepressant use also increased with age. Investments in pharmaceutical services are needed to monitor the rational use of antidepressants in the Brazilian population, especially in vulnerable individuals, such as older adults. Future studies may also elucidate the therapeutic subgroups of antidepressants most often used by Brazilians and their correlations with psychiatric diagnoses.

## Disclosure

The authors report no conflicts of interest.

## Figures and Tables

**Table 1:** Characteristics of the studies included in the systematic review

Study	Study year	Region	Study design	Population (years old)	Mean age (years)	Sample size	Recall period (days)	Coding system
Abi-Ackel^13^	2003	Southeast	Cross-sectional	Older adults (≥ 60)	69.7	1,635	15	ATC
Bertoldi^14^	2002	South	Cross-sectional	Adults ≥ 20	32.9	3,182	15	Brazilian List of Essential Medicines
Blay^15^	2002	Southeast	Cross-sectional	Adults 18-65	NR	2,000	360	Ministry of Health List of Controlled Products
Campanha^16^	2005-2007	Southeast	Cross-sectional	Adults ≥ 18	41.0	2,935	360	ATC
Fernandes^17^	2014-2015	Southeast	Cross-sectional	Adults ≥ 20	54.4	1,953	15	ATC
Garcias^18^	2006	South	Cross-sectional	Adults ≥ 40	NR	1,327	15	Coding system not reported or not used
Lima^19^	1994	South	Cross-sectional	Any ≥ 15	41.4	1,277	15	Ministry of Health List of Controlled Products
Lima^20^	2001-2002	Southeast	Cross-sectional	Any ≥ 15	49.0	1,023	3	ATC
Loyola Filho^21^	1997 and 2012	Southeast	Cohort	Older adults (≥ 60)	1997: 79.82012: 79.9	1997: 3512012: 462	Day of the interview	ATC
Loyola Filho^22^	2021	Southeast	Cross-sectional	Adults ≥ 18	NR	2,805	15	ATC
Mari^23^	1990	Southeast	Cross-sectional	Any ≥ 14	NR	1,742	360	Coding system not reported or not used
Menolli^24^	2011 and 2015	South	Cohort	Adults ≥ 40	54.2	2011: 1,1802015: 885	Day of the interview	ATC
Moraes^25^	2012-2013	Southeast	Cross-sectional	Adult women 45-60	52.5	749	Day of the interview	Coding system not reported or not used
Neves^26^	2014	North	Cross-sectional	Adults 18-59	NR	685	30	ATC
Noia^27^	2006	Southeast	Cross-sectional	Older adults (≥ 60)	NR	1,115	Day of the interview	ATC
Pinto^28^	2007	Southeast	Cross-sectional	Adults ≥ 18	NR	423	Day of the interview	ATC
Prado^29^	2008-2009	Southeast	Cross-sectional	Adults ≥ 20	42.7	2,472	3	ATC
Quintana^30^	2007-2008	Southeast	Cross-sectional	Any ≥ 15	42.0	1,208	30 and 360	Coding system not reported or not used
Quintana^31^	2007	Southeast	Cross-sectional	Any 15-75	39.5	2,356	30	Coding system not reported or not used
Rodrigues^32^	1994 and 2003	South	Cross-sectional	Any ≥ 15	NR	3,542	15	Ministry of Health List of Controlled Products
Rodrigues^33^	2013-2014	Brazil	Cross-sectional	Adults ≥ 20	NR	32,348	15	European Study of the Epidemiology of Mental Disorders
Tiguman^34^	2015 and 2019	North	Cross-sectional	Adults ≥ 18	2015: 38.42019: 37.3	2015: 3,4792019: 2,321	15	ATC
Vicente^35^	1997	Southeast	Cohort	Older adults (≥ 60)	69.3	1,606	90	ATC

ATC = Anatomical Therapeutic Chemical classification; NR = not reported/no response from authors.

**Table 2:** Prevalence (95%CI) and magnitude of inconsistency (*I*^2^) of antidepressant use in Brazil by sex and age group according to the recall period

	Adults			Older adults			Older adults vs.adults	Women			Men			Women vs.men
Recall period (days)	Sample size (no. of studies)	% (95%CI)	*I*^2^ (%)	Sample size (no. of studies)	% (95%CI)	*I*^2^ (%)	p-value (Q)	Sample size (no. of studies)	% (95%CI)	*I*^2^ (%)	Sample size (no. of studies)	% (95%CI)	*I*^2^ (%)	p-value (Q)
0	2,488 (3)	5.6 (1.8-11.3)	96.1	1,928 (3)	12.2 (4.3-23.4)	97.3	0.225 (1.47)	749 (1)	14.7 (12.3-17.4)	-	- (0)	-	-	-
3	1,023 (1)	5.0 (3.8-6.5)	-	- (0)	-	-	-	525 (1)	12.0 (9.5-15.1)	-	498 (1)	4.6 (3.1-6.8)	-	< 0.001 (18.68)
15	45,887 (7)	3.5 (1.7-5.9)	98.9	11,260 (4)	4.6 (1.8-8.6)	97.5	0.590 (0.29)	4,646 (3)	4.6 (0.0-19.5)	0.0	3,999 (3)	2.1 (0.0-7.4)	0.0	0.638 (0.22)
30	3,564 (2)	2.8 (2.0-3.9)	0.0	- (0)	-	-	-	2,124 (2)	4.3 (3.5-5.2)	0.0	1,620 (2)	1.1 (0.6-1.7)	0.0	< 0.001 (38.38)
90	- (0)	-	-	1,606 (1)	8.4 (7.1-9.9)	-	-	965 (1)	11.4 (9.5-13.6)	-	641 (1)	3.9 (2.7-5.7)	-	< 0.001 (33.49)
360	7,885 (4)	2.7 (0.5-6.4)	98.4	- (0)	-	-	-	1,581 (2)	2.3 (1.6-3.1)	37.6	1,369 (2)	0.5 (0.2-1.0)	0.0	< 0.001 (17.23)

## Data Availability

The datasets of the present study are openly available at https://osf.io/v8trp/.
