# Supplementary material for: Prevalence of antidepressant use in Brazil: a systematic review with meta-analysis
Source: Braz J Psychiatry. 2024 Mar 25;46:e20233095. doi: 10.47626/1516-4446-2023-3095 (PMC11189131; doi:10.47626/1516-4446-2023-3095)
Supplement: Supplementary file 1 [file bjp-46-e20233095-s001.pdf]

http://doi.org/10.47626/1516-4446-2023-3095

Figure S1 Odds ratio of antidepressant use in women versus men according to the recall period

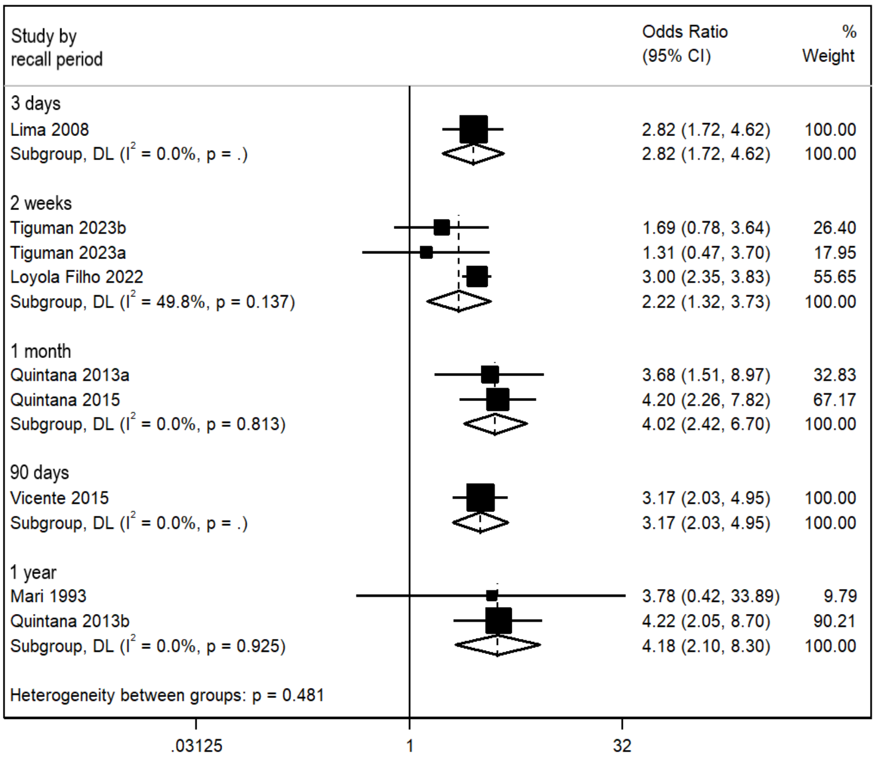

Figure S2 Prevalence of antidepressant use according to the standard error of prevalence

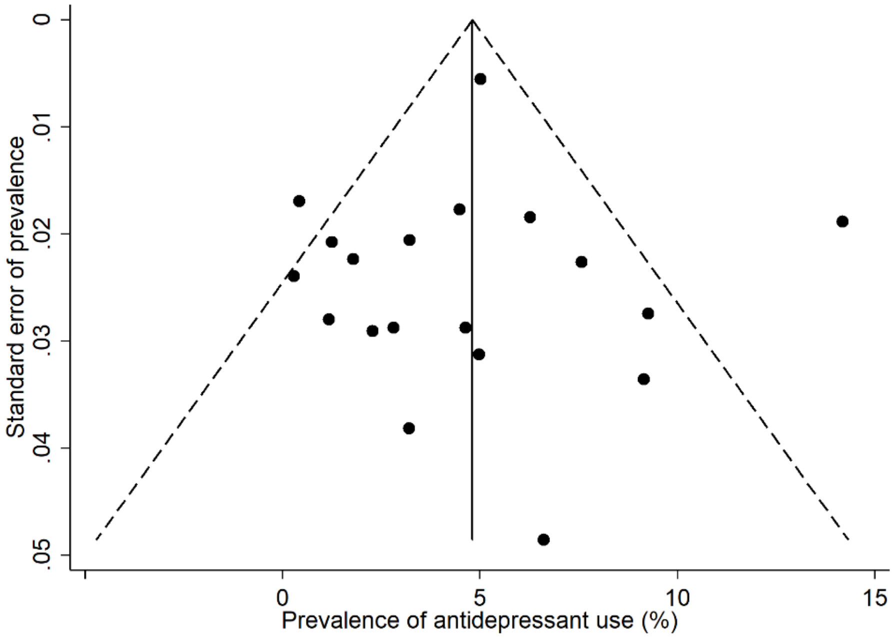

Table S1 Search strategies employed (search last conducted in May 2023)

Database	Search strategy
PubMed	((((Antidepressive Agents) OR (Antidepressant Drug) OR Antidepressants OR Antidepressant OR (Antidepressant Drugs) OR (Antidepressant Medication) OR (Antidepressive Agent) OR escitalopram OR sertraline OR amitriptyline OR desvenlafaxine OR duloxetine OR venlafaxine) OR ((Psychotropic Drugs) OR (Psychoactive Agent) OR (Psychoactive Drug) OR Psychopharmaceuticals OR (Psychoactive Agents) OR (Psychoactive Drugs) OR (Psychotropic Drug) OR Psychopharmaceutical)) AND (Prevalence OR Prevalences OR (Period Prevalence) OR (Period Prevalences) OR (Point Prevalence) OR (Point Prevalences) OR (Observational Study) OR (Cross-Sectional Study) OR (Cross-Sectional Studies) OR (Cohort Study) OR (Cohort Studies) OR (case-control study) OR (case-control studies) OR (population based) OR (retrospectiv*) OR (prospectiv*))) AND (Brazil* OR Brasil*)
EMBASE	#6<br>(#1 OR #2) AND #3 AND #4 NOT #5<br>1,006<br>#5<br>[medline]/lim OR [pubmed-not-medline]/lim<br>30,242,585<br>#4<br>'brazil'/exp OR 'brasil*' OR 'brazil*'<br>765,752<br>#3<br>'prevalence'/exp OR 'prevalence study' OR 'observational study'/exp OR 'cross-sectional study'/exp OR 'cohort analysis'/exp OR 'case control study'/exp OR 'retrospective study'/exp OR 'prospective study'/exp<br>3,698,676<br>Edit<br>Email alert<br>RSS feed<br>#2<br>'psychotropic agent'/exp OR 'psychotropic agent' OR 'psychotropic' OR 'psychopharmaceutic'<br>1,140,902<br>#1<br>('antidepressant agent'/exp OR antidepressive) AND agent OR 'escitalopram'/exp OR 'sertraline'/exp OR 'amitriptyline'/exp OR 'desvenlafaxine'/exp OR 'duloxetine'/exp OR 'venlafaxine'/exp
SCOPUS	(TITLE-ABS-KEY ("antidepressive agent" OR "antidepressant agent"' OR "antidepressant" OR "escitalopram" OR "sertraline" OR "amitriptyline" OR "desvenlafaxine" OR "duloxetine" OR "venlafaxine") OR TITLE-ABS-KEY ("psychotropic agent" OR "psychotropic" OR "psychopharmaceutic")) AND TITLE-ABS-KEY ("prevalence" OR "prevalence study" OR "observational study" OR "cross-sectional study" OR "cohort analysis" OR "case control study" OR "retrospective study" OR "prospective study") AND TITLE-ABS-KEY (brazil OR brasil OR brasil* OR brazil*)
SciELO	((antidepressive agent) OR (antidepressant agent) OR (antidepressant) OR (escitalopram) OR (sertraline) OR (amitriptyline) OR (desvenlafaxine) OR (duloxetine) OR (venlafaxine) OR (psychotropic agent) OR (psychotropic) OR (psychopharmaceutic)) AND ((prevalence) OR (prevalence study) OR (observational study) OR (cross-sectional study) OR (cohort analysis) OR (case control study) OR (retrospective study) OR (prospective study)) AND ((brazil) OR (brasil))
LILACS (via BVS)	((antidepressive agent) OR (antidepressant agent) OR (antidepressant) OR (escitalopram) OR (sertraline) OR (amitriptyline) OR (desvenlafaxine) OR (duloxetine) OR (venlafaxine) OR (psychotropic agent) OR (psychotropic) OR (psychopharmaceutic)) AND ((prevalence) OR (prevalence study) OR (observational study) OR (cross-sectional study) OR (cohort analysis) OR (case control study) OR (retrospective study) OR (prospective study)) AND (brazil* OR brasil*) AND (db:("LILACS"))

Table S2 Criteria for assessing the methodological quality of studies according to the Joanna Briggs Institute checklist for prevalence studies

Item	Criteria
1. Sample frame	Sample was representative of the general population in Brazil.
2. Sampling method	Selection of participants was done using probabilistic or universal sampling.
3. Sample size	Sample size was statistically calculated.
4. Description of participants	Characteristics of participants were adequately described.
5. Data coverage	Data analysis conducted with sufficient coverage in each group.
6. Valid methods for the outcome	Medical prescriptions or drug packages were confirmed and a reliable coding system was used for classification of the antidepressants.
7. Outcome measurement	Outcome measurement was standardized and uniformly assessed in all participants.
8. Statistical analysis	Numerators and denominators of antidepressant use, or measures of dispersion, were available.
9. Response rate	Losses did not impact the outcome or over 70% of response rate.

http://doi.org/10.47626/1516-4446-2023-3095

Table S3 Quality appraisal of the studies included in the systematic review

Study	Source	Sampling	Study size	Description	Coverage	Outcome	Measurement	Analysis	Response	Total
Abi-Ackel 2017[13]	1	1	1	1	1	1	1	1	1	9
Bertoldi 2004[14]	1	1	1	1	1	1	1	1	1	9
Blay 2014[15]	1	1	1	1	1	0[†]	1	1	0[‡]	8
Campanha 2015[16]	1	1	1	1	1	0[†]	1	1	1	7
Fernandes 2018[17]	1	1	1	1	1	1	1	1	1	9
Garcias 2008[18]	1	1	1	1	1	0[†]	1	1	1	8
Lima 1999[19]	1	1	1	0[§]	1	1	1	1	1	8
Lima 2008[20]	1	1	1	1	1	1	1	0[‖]	1	8
Loyola Filho 2014[21]	1	1	1	1	1	1	1	0[‖]	1	8
Loyola Filho 2022[22]	1	1	1	1	1	0[†]	1	0[‖]	1	7
Mari 1993[23]	0[¶]	1	0[††]	1	0[¶]	0[†]	0[‡‡]	0[‖]	1	3
Menolli 2020[24]	1	1	1	1	1	1	1	1	1	9
Moraes 2019[25]	1	1	1	1	1	1	1	0[‖]	1	8
Neves 2022[26]	1	1	1	0[§]	0[¶]	1	1	0[‖]	1	6
Noia 2012[27]	1	1	0[††]	0[§]	1	0[†]	0[‡‡]	0[‖]	0[§§]	3
Pinto 2012[28]	1	1	1	0[§]	0[¶]	0[†]	0[‡‡]	0[‖]	0[§§]	3
Prado 2017[29]	1	1	1	1	1	1	1	1	1	9
Quintana 2013[30]	1	1	1	1	1	0[†]	1	0[‖]	1	7
Quintana 2015[31]	1	1	1	1	1	0[†]	1	1	1	8

http://doi.org/10.47626/1516-4446-2023-3095

Rodrigues 2006[32]	1	1	1	1	1	1	1	0[‖]	1	8
Rodrigues 2020[33]	1	1	1	1	1	0[†]	1	0[‖]	0[§§]	6
Tiguman 2023[34]	1	1	1	1	1	0[†]	1	1	1	8
Vicente 2015[35]	1	1	1	1	1	1	1	1	1	9
Total	22	23	21	19	20	12	20	12	19	-

[†] Confirmation was not reported, or partial confirmation was performed for some of the drug packages/prescriptions, or drugs were not classified using a coding system.
[‡] Response rate not reported.
[§] Description of the participants' characteristics was absent or too limited to allow generalization of the results.
[‖] Numerators and denominators and/or measures of dispersion of antidepressant use were not reported.
[¶] Sampling was not representative of the city and/or coverage of the analysis was restricted.
[††] Sample size calculations were not reported.
[‡‡] Method of outcome measurement was not reported.
[§§] Response rate was < 70%.
